# Myanmar *Burkholderia pseudomallei* strains are genetically diverse and originate from Asia with phylogenetic evidence of reintroductions from neighbouring countries

**DOI:** 10.1038/s41598-020-73545-8

**Published:** 2020-10-01

**Authors:** Jessica R. Webb, Mo Mo Win, Khwar Nyo Zin, Kyi Kyi Nyein Win, Thin Thin Wah, Elizabeth A. Ashley, Frank Smithuis, Myo Maung Maung Swe, Mark Mayo, Bart J. Currie, David A. B. Dance

**Affiliations:** 1 Global and Tropical Health Division, Menzies School of Health Research, Charles Darwin University, Darwin, NT Australia; 2 Department of Infectious Diseases and Northern Territory Medical Program, Royal Darwin Hospital, Darwin, NT Australia; 3 Department of Medical Research, Yangon, Myanmar; 4 Centre for Tropical Medicine and Global Health, Nuffield Department of Clinical Medicine, University of Oxford, Old Road Campus, Oxford, UK; 5 Lao-Oxford-Mahosot Hospital-Wellcome Trust Research Unit, Microbiology Laboratory, Mahosot Hospital, Vientiane, Laos; 6 Myanmar-Oxford Clinical Research Unit, Yangon, Myanmar; 7 Faculty of Infectious and Tropical Diseases, London School of Hygiene and Tropical Medicine, London, UK; 8 Microbiology Laboratory, Yangon General Hospital, Yangon, Myanmar

**Keywords:** Computational biology and bioinformatics, Evolution, Genetics, Molecular biology

## Abstract

Melioidosis was first identified in Myanmar in 1911 but for the last century it has remained largely unreported there. *Burkholderia pseudomallei* was first isolated from the environment of Myanmar in 2016, confirming continuing endemicity. Recent genomic studies showed that *B. pseudomallei* originated in Australia and spread to Asia, with phylogenetic evidence of repeated reintroduction of *B. pseudomallei* across countries bordered by the Mekong River and the Malay Peninsula. We present the first whole-genome sequences of *B. pseudomallei* isolates from Myanmar: nine clinical and seven environmental isolates. We used large-scale comparative genomics to assess the genetic diversity, phylogeography and potential origins of *B. pseudomallei* in Myanmar. Global phylogenetics demonstrated that Myanmar isolates group in two distantly related clades that reside in a more ancestral Asian clade with high amounts of genetic diversity. The diversity of *B. pseudomallei* from Myanmar and divergence within our global phylogeny suggest that the original introduction of *B. pseudomallei* to Myanmar was not a recent event. Our study provides new insights into global patterns of *B. pseudomallei* dissemination, most notably the dynamic nature of movement of *B. pseudomallei* within densely populated Southeast Asia. The role of anthropogenic influences in both ancient and more recent dissemination of *B. pseudomallei* to Myanmar and elsewhere in Southeast Asia and globally requires further study.

## Introduction

The opportunistic environmental biothreat agent *Burkholderia pseudomallei* causes melioidosis, a disease of diverse manifestations with mortality rates ranging from 9 to 40%^1,2^. Melioidosis was first described in Rangoon (now Yangon: 21.9° N, 95.9° E), Myanmar (formerly Burma) in 1911 by British pathologist, Alfred Whitmore, in a 40-year-old morphine addict who died from a fever associated with multiple subcutaneous abscesses and pneumonia^3,4^. Over the next 5 years, Whitmore’s assistant Krishnaswamy saw over 200 cases of what was then known as ‘morphia injectors’ septicaemia’ in Rangoon^5^. Since then, however, reports of melioidosis in Myanmar have dwindled and become sporadic, with only a handful of cases reported in the 1940s^6,7^ before the disease was rediscovered more than half a century later, in 2000 in Mandalay^8^. The gradual re-emergence of melioidosis in Myanmar has recently been reviewed^6^.

The climate in Myanmar varies from north to south but is generally tropical, with rainfall varying from 5500 mm in the coastal regions to less than 1000 mm in the central ‘Dry Zone’^9^. Recent modelling predicted that the environment of much of Myanmar would be suitable for the persistence of *B. pseudomallei*^10^, although the organism was only first detected in environmental samples from Myanmar in 2016, more than a century after its initial description by Whitmore. The diagnosis of culture-confirmed human melioidosis cases at Yangon General Hospital in 2009 prompted soil sampling on two farms in nearby townships, Hmawbi and Thanlyin. *B. pseudomallei* was isolated from seven soil samples: five from Hmawbi and two from Thanlyin^11^. Subsequently a systematic survey of the environment in Myanmar was initiated in 2018 in order to identify areas of high melioidosis risk (Smithuis, manuscript in preparation).

Although melioidosis was initially discovered in Myanmar, at the time of initiating this study there were no isolates of *B. pseudomallei* included in the international Multilocus Sequence Typing (MLST) database (https://pubmlst.org/bpseudomallei/) to inform understanding of the place of the country in the global phylogeography of the species. Prior studies have shown that *B. pseudomallei* originally evolved in the environment of Australia and subsequently spread to Southeast Asia and East Asia during the last glacial period (between 16 and 225 thousand years ago)^12^. From Asia, *B. pseudomallei* subsequently spread to Madagascar and Africa and more recently from West Africa to the Americas (between 1650 and 1850), likely linked to the slave trade^12–15^. This strong geographic signal of the *B. pseudomallei* genome is notably due to infection in humans and animals being acquired directly from an environmental source, with human–human and zoonotic transmission being exceedingly rare. Such epidemiology and large continental geographic barriers restrict gene flow and have resulted in distinct geographic populations of *B. pseudomallei*.

The isolation of *B. pseudomallei* from patients and the environment in Myanmar not only confirmed the continuing endemicity of melioidosis in the country but enabled phylogenetic comparisons of *B. pseudomallei* from Myanmar with those from neighbouring countries and the global *B. pseudomallei* dataset in order to infer the origins and investigate whether the recent re-emergence of melioidosis in Myanmar reflected persistence or recent re-introductions. This study included sixteen *B. pseudomallei* isolates from Myanmar (nine human and seven environmental). We present the first Myanmar *B. pseudomallei* whole-genome sequences (WGS) and, using large-scale comparative-genomics, we attempt to determine the origin of *B. pseudomallei* in Myanmar.

## Methods

### Study setting and Myanmar *B. pseudomallei* isolates

**Figure 1:**
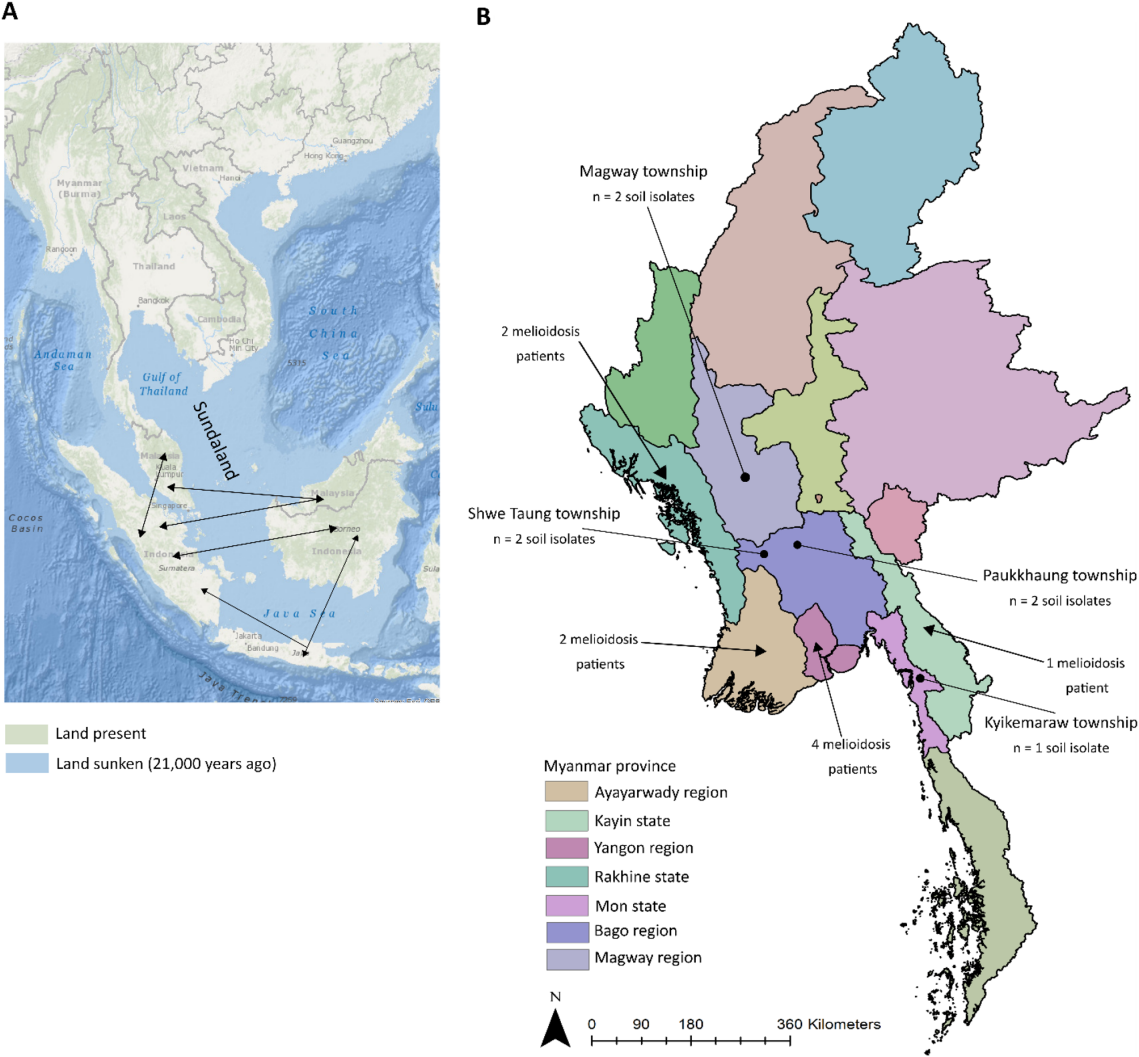
**(A)** Map of Sundaland region, demonstrating when a larger landmass was exposed in the region (including light blue and green shading) and after the Sunda shelf was inundated by rising sea levels (green shading only). Black arrows indicate potential dispersal routes for terrestrial organisms^16^. **(B)** Myanmar and its 14 regions, showing where *B. pseudomallei* was isolated from soil and regions where the nine melioidosis patents resided. Maps were created using ArcGIS V10.4.1 (https://www.arcgis.com/index.html).

Myanmar has a total land area of 676,577 km^2^, with a length of 2,090 km from north to south, has four important river systems, with the Ayeyarwaddy River the main waterway, and comprises 14 states and regions. It extends south towards the Malay Peninsula, is bordered by China to the north and northeast, Laos to the east, Thailand to the south and east, Bangladesh to the west and India to the northwest and is situated within the biogeographical region of Sundaland (Fig. 1A).

Included in this study were sixteen *B. pseudomallei* isolates from Myanmar collected from 2017 to 2018 during ongoing enhanced surveillance for clinical cases of melioidosis and the national environmental survey. The collection comprised seven soil isolates from Kyaikmaraw township, Mon state (one isolate), Paukkhaung township, Bago region (two isolates), Shwe Taung township, Bago region (two isolates) and Magway township, Magway region (two isolates) (Fig. 1B), and nine isolates from melioidosis patients in Ayeyarwady region (two patients), Kayin state (one patient), Yangon region (four patients) and Rakhine state (two patients) (Fig. 1). All patients were believed to have acquired their infections in Myanmar with no history of travel. None of the soil isolates were epidemiologically linked to the melioidosis patients.

### Sequencing, quality control and assembly

Culture of *B. pseudomallei* was performed using methods previously established^17–19^ and their identity was confirmed using a real-time PCR assay targeting a *B. pseudomallei*-specific 115-bp segment within the type three secretion system 1 (TTS1) gene^20^. Genomic DNA was extracted from purified *B. pseudomallei* colonies as previously described^21^. WGS were performed on the Illumina NovaSeq platform, with the sequencing platform generating 150 bp paired-end reads for each genome. Genomic analysis included an additional 173 publicly available *B. pseudomallei* genomes and all genomes are available on the sequence read archive database (Table S1). Read quality was conducted using Trimmomatic v0.39^22^ and FastQC (https://www.bioinformatics.babraham.ac.uk/projects/fastqc) and then assembled using the MGAP pipeline (https://github.com/dsarov/MGAP-Microbial-Genome-Assembler-Pipeline) generating high quality draft assemblies (contigs ≤ 90 and N50 > 180,000 bp).

### MLST assignment

MLST was performed to assign STs to isolates by determining allele sequence in silico from bacterial WGS read data using the BIGSdb tool, which is accessible on the *B. pseudomallei* MLST website (https://pubmlst.org/bpseudomallei/).

### Variant calling, phylogenetic analysis, recombination identification and pan-genome analysis

Single-nucleotide polymorphisms (SNP) and small insertions/deletion (Indels) variants were identified from WGS data using Genome Analysis Toolkit (GATK v4.1.0.0) (https://github.com/broadinstitute/gatk) wrapped in SPANDx v3.2^23^ and the closed K96243 (accessions BX571965 and BX571966) genome was used as the reference for all phylogenetic analysis. The SNP and Indel variants identified by GATK were used for phylogenetic reconstruction using maximum parsimony (MP) in PAUP* 4.0.b5^24^ or SNP variants for maximum likelihood (ML) in RAxML^25^. Bootstrapping using 1000 replicates was carried out for MP and phylogenetic trees were visualised and annotated using Interactive Tree of Life (ITOL) (https://itol.embl.de). Regions of recombination were predicted using Gubbins v2.3.4 (Genealogies Unbiased by Recombination In Nucleotide Sequences, v.2.3.1) (https://github.com/sanger-pathogens/gubbins) with default settings. The High-quality isolate assemblies were annotated using Prokka (v1.13) and proteomes were used for pan-genomic analysis, which was calculated for the 16 Myanmar isolates using Roary (v3.12.0)^26^. Detection of virulence genes (LPS A (*wbil* to *apaH* in K96243 [GenBank ref: NC_006350]), LPS B (*BUC_3392* to *apaH* in *B. pseudomallei* 576 [GenBank ref: NZ_ACCE01000003]), LPS B2 (*BURP840_LPSb01* to *BURP840_LPSb21* in *B. pseudomallei* MSHR840 [GenBank ref: GU574442]), *bimA*_*Bm*_ (*BURPS668_A2118* in *B. pseudomallei* MSHR668 [GenBank ref: NZ_CP009545]) *bimA*_*Bp*_ (*BPSS1492* in *B. pseudomallei* K96243 [GenBank ref: NC_006351.1]) and *fhaB3* (*BPSS2053* in *B. pseudomallei* K96243 [GenBank ref: NC_006351.1])) was determined using the Large scale BLAST score ratio (LS-BSR) pipeline with standard settings and blastn or tblastn^27^. We used a BSR threshold of > 0.9 for presence of virulence genes and scores below the cut-off were deemed absent or variable.

## Results

### Myanmar *B. pseudomallei* isolates are genetically diverse

**Figure 2:**
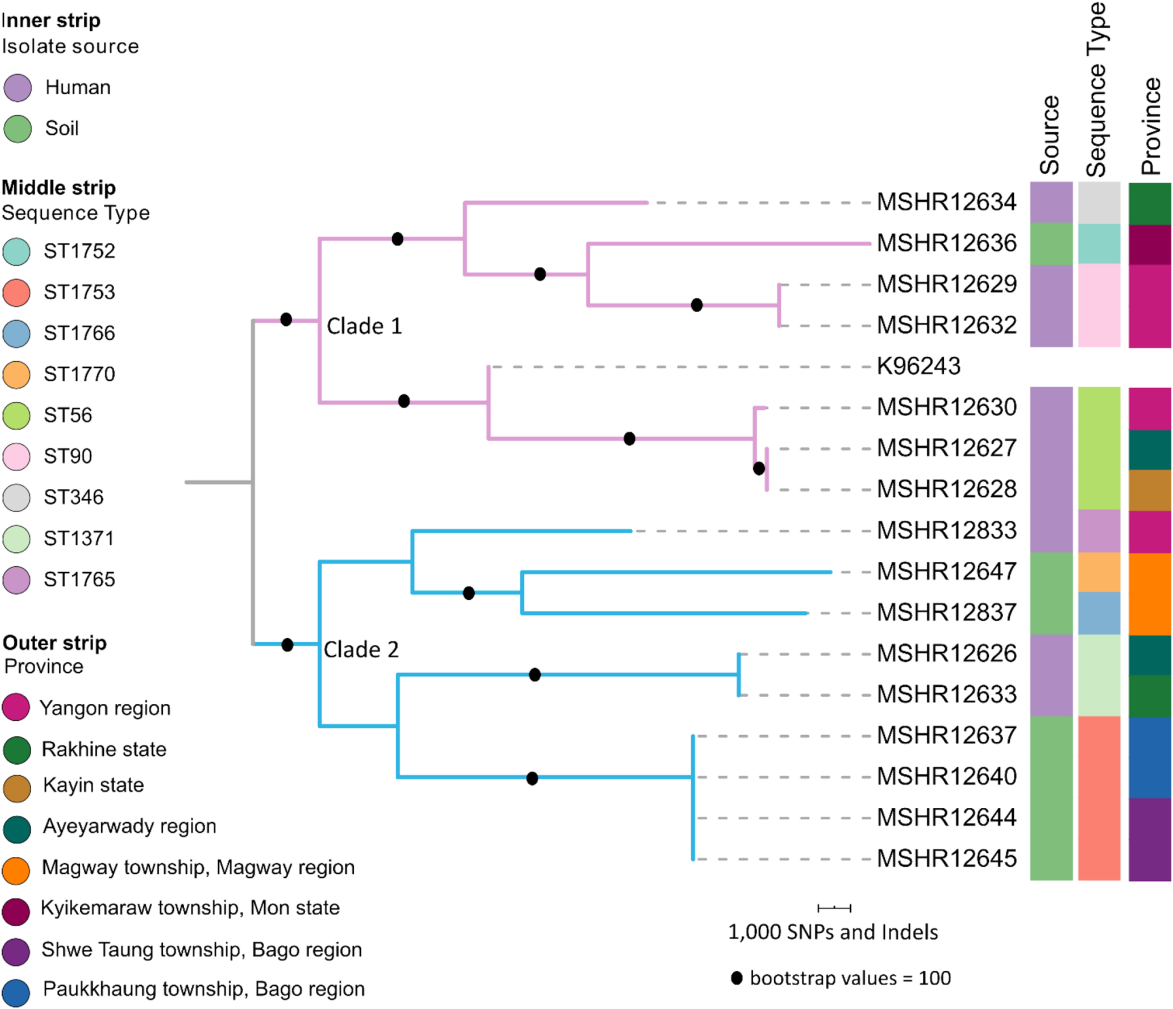
Midpoint rooted maximum parsimony phylogeny of *B. pseudomallei* (n = 16) from Myanmar, based on 60,204 core genome SNPs-indels.

In silico MLST of the 16 Myanmar genomes identified nine STs [ST56 (n = 3), ST90 (n = 2), ST346 (n = 1), ST1371 (n = 2), ST1752 (n = 1), ST1753 (n = 4), ST1765 (n = 1), ST1766 (n = 1) and ST1770 (n = 1)] and of these nine STs, five STs were novel and so far only found in Myanmar (Table 1). ST1753 was the only ST identified from the environment in more than one location, being identified in two towns in the Bago region separated by ~ 50 km. MLST revealed that none of the STs of the clinical isolates matched the STs of the environmental isolates, with phylogenetics confirming that the environmental isolates recovered from Myanmar in this study were not closely related to the patient isolates, with > 18,000 SNPs-indels separating clinical isolates from environmental isolates (Fig. 2). This was not unexpected as the soil isolates were not specifically collected in locations linked to the individual cases of melioidosis.

**Table 1:** Myanmar isolates included in this study.

Isolate	Aliases	Year	Myanmar province	Below surface (cm)	Sample ID	ST	FhaB3	LPS type	BimA type
**Human**									
MSHR12626	Y3	2017	Ayeyarwady region	N/A	N/A	1371	Negative	LPS B	BimA_Bp_
MSHR12627	Y21	2017	Ayeyarwady region	N/A	N/A	56	Negative	LPS A	BimA_Bp_
MSHR12628	Y22	2017	Kayin state	N/A	N/A	56	Negative	LPS A	BimA_Bp_
MSHR12629	Y42	2018	Yangon region	N/A	N/A	90	Positive	LPS A	BimA_Bp_
MSHR12630	M72	2018	Yangon region	N/A	N/A	56	Negative	LPS A	BimA_Bp_
MSHR12632	M92	2018	Yangon region	N/A	N/A	90	Positive	LPS A	BimA_Bp_
MSHR12833	MM73014	2018	Yangon region	N/A	N/A	1765*	Positive	LPS A	BimA_Bp_
MSHR12633	Y43	2018	Rakhine state	N/A	N/A	1371	Negative	LPS B	BimA_Bp_
MSHR12634	Y33	2018	Rakhine state	N/A	N/A	346	Positive	LPS A	BimA_Bp_
**Environmental**									
MSHR12636	755	2018	Kyikemaraw township, Mon state	60	MON 20-005	1752*	Positive	LPS A	BimA_Bp_
MSHR12637	2119	2018	Paukkhaung township, Bago region	90	BGO 02-009	1753*	Positive	LPS B	BimA_Bp_
MSHR12640	2128	2018	Paukkhaung township, Bago region	90	BGO 03-008	1753*	Positive	LPS B	BimA_Bp_
MSHR12644	2139	2018	Shwe Taung township, Bago region	90	BGO 04-009	1753*	Positive	LPS B	BimA_Bp_
MSHR12645	2143	2018	Shwe Taung township, Bago region	30	BGO 05-003	1753*	Positive	LPS B	BimA_Bp_
MSHR12647	2844	2018	Magway township, Magway region	60	MGY 16-004	1770*	Positive	LPS A	BimA_Bp_
MSHR12837	2897	2018	Magway township, Magway region	60	MGY 21-007	1766*	Positive	LPS A	BimA_Bp_

Symbol: *novel ST; N/A not applicable/not available.

On interrogating the *B. pseudomallei* PubMLST database, four of the nine STs were shared by neighbouring countries: ST56, Bangladesh (seven isolates), Cambodia (two isolates), Thailand (four isolates) and Vietnam (two isolates); ST90, United Kingdom with history of travel to Asia (eleven isolates), a USA isolate with unknown travel history, China (one isolate); ST1371, India (two isolates) and ST346, France (one isolate). The original source of the isolates linked to the United Kingdom, USA and France, which were presumably imported, is not available. Draft genomes from three ST56 genomes, one from Bangladesh and two from Vietnam were available for comparison to our Myanmar ST56s (see next section).

Comparative genomics revealed that the Myanmar isolates were genetically diverse with 60,204 core genome SNPs and indels identified among the 16 Myanmar genomes (Fig. 2). The SNPs and indels were used to construct a Myanmar-only phylogenetic tree, revealing two distinct clades: clade one (seven isolates) and clade two (nine isolates) which were separated by 4157 SNPs-indels (Fig. 2). There was some evidence of phylogenetic structure, with environmental isolates from the same province clustered together (Fig. 2). For example, the four ST1753 isolates from Bago region (Shwe Taung and Paukkhaung) clustered together on clade two and were genetically similar with two SNPs-indels being the largest genetic difference in the ST1753 cluster. There were instances where isolates from the same region clustered together but were genetically dissimilar and belonged to different STs, with two soil isolates from Magway region clustering together but separated by 18,464 SNPs-indels.

For the clinical isolates, isolates belonging to the same ST clustered together, but in most instances corresponding patients resided in different states/regions. For example, three ST56 clinical isolates (MSHR12630, MSHR12627 and MSHR12628) clustered together with the greatest genetic difference of six SNPs-indels, but patients resided in; Yangon region, Kayin state or Ayeyarwaddy region with the greatest distance difference of 291 km. In another example, two clinical isolates belonging to ST1371 differed by only four SNPs-indels but the corresponding patients resided many kilometres apart in Rakhine state and Ayeyarwaddy region. There was one instance where clinical isolates clustered together based on ST and geography: ST90 clinical isolates from two patients residing in Yangon region clustered together were separated by only five SNPs-indels.

We found considerable evidence of recombination among the 16 Myanmar isolates, (Figure S1B; recombination events are represented by the colour blocks) although removing regions associated with recombination did not significantly alter the phylogenetic structure of the isolates (Figure S1A).

### Phylogenetic analysis reveals reintroductions into Myanmar from neighbours

**Figure 3:**
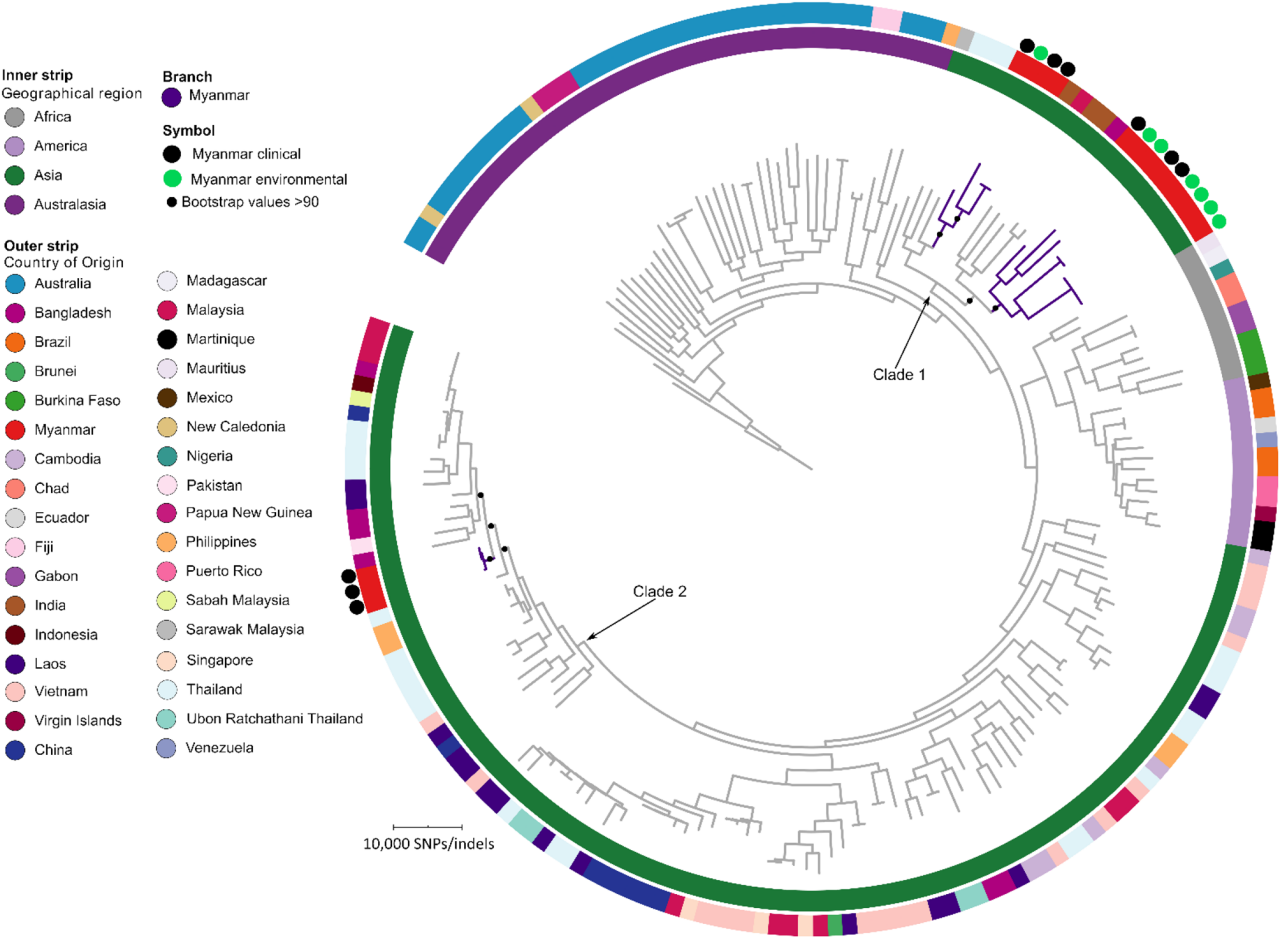
Maximum parsimony phylogeny of *B. pseudomallei* from Myanmar (n = 16) with a global set of genomes (n = 173) based on 167,781 core genome SNPs-indels. Rooted with MSHR0668, the most ancestral *B. pseudomallei* strain, as identified in a large phylogenetic study^29^.

Comparative analysis of the 16 Myanmar *B. pseudomallei* genomes with a global set of 173 genomes, identified 168,934 core genome SNPs-indels, which were used to construct a phylogenetic tree to identify the origin of *B. pseudomallei* from Myanmar. The Myanmar isolates (purple branches, Fig. 3) resided in two clades within the Asian clade. For the purpose of this study we defined clades containing Myanmar isolates as clade one and clade two (Fig. 3). Myanmar isolates located on clade one were the most ancient given their proximity to the ancient Australian isolates located at the tree base, and clade two was located further away from the tree base so these isolates were the least ancient. Clade one isolates had the longest branches out of the Myanmar isolates and so were the most diverse, with Myanmar isolates in clade two the least diverse with the shortest branches.

Phylogeography demonstrated that the Myanmar isolates shared nodes and clustered with isolates from neighbouring countries (Fig. 3). Clade one (thirteen Myanmar isolates); four Myanmar isolates shared a node with four Thai isolates (moderate bootstrap value 70%) and nine Myanmar isolates shared a node with a cluster that contained isolates from India (three isolates), Bangladesh (one isolate) and Malaysia (one isolate) (bootstrap value 92%). Clade two (three ST56 Myanmar isolates): three Myanmar isolates shared a node with a cluster that contained isolates from Bangladesh (four isolates), Pakistan (one isolate: the true origin of this isolate is uncertain^28^), Laos (two isolates), Thailand (four isolates), China (one isolate), Malaysia (four isolates) and Indonesia (one isolate) (well supported bootstrap value 97%), and descended from a Thai isolate. The closest isolate to a Myanmar isolate was a Thai isolate in clade two which differed by 4,317 SNPs-indels from a Myanmar isolate and the node was well supported (bootstrap value 97%). An ML global phylogeny supported the clustering observed here as did an Asian-only phylogeny (data not shown).

The ST56 isolates from Vietnam, Myanmar and Bangladesh were located in the Asian clade, but were distantly related to one another. The Myanmar clade containing the ST56s was separated from the ST56 from Bangladesh by 5,688 SNPs-indels (SID2889) and the ST56s from Vietnam by 12,102 SNPs-indels (MSHR6971) or 12,103 SNPs-indels (MSHR6972). Global phylogenetics demonstrated that the ST56 isolates from Vietnam were located nearest to the tree base, followed by ST56 isolates from Myanmar, with the ST56 isolate from Bangladesh the furthest away from the tree base.

### Gene content of *B. pseudomallei* isolates from Myanmar

The virulence genes screened for in the Myanmar isolates varied. For BimA: all 16 Myanmar isolates contained BimA, *bimA*_*Bp*_, for *fhaB3*: 11 (69%) isolates contained *fhaB3* and five (31%) lacked the gene, for LPS type: 10 (63%) isolates contained LPS A and the remaining six (37%) isolates contained LPS B. LPS A and LPS B were associated with different regions, with all environmental LPS B isolates from Bago region and LPS A from Magway region or Mon state (Table 1).

For the Myanmar pan-genome, which was calculated for the 16 isolates using Roary, the accessory genome contained heterogeneity indicating gene diversity among the Myanmar isolates, but the core genome (genes occurring in > 99% of isolates) was stable (Figure S2). We identified a total of 9246 predicted coding sequences (CDS) with 5249 and 3997 assigned to the core and accessory genome. The number of core genes was comparable to that reported previously, but the number of accessory genes was greater than previously reported, (based on 37 *B. pseudomallei* genomes ^30^) highlighting the high genetic diversity of the Myanmar isolates.

## Discussion

The scarceness of genotype and phylogenetic overlap between Australian and Asian *B. pseudomallei* is largely due to ancient oceanic biogeographical barriers, including Wallace’s Line, restricting gene flow between populations^31^. The same cannot be said about *B. pseudomallei* isolates originating within Asia. Southeast Asian countries bordered by the Mekong River and the Malay Peninsula have shared genotypes and phylogenetic overlap^12^; this is likely due to the lack of geographical barriers, both as exist currently, but even more extensively during the last ice age within an expanded Sundaland (Fig. 1A). Here we present the first genomic analysis of *B. pseudomallei* from Myanmar, where melioidosis was first identified in 1911. Given that Myanmar shares geographical borders with its Southeast Asian neighbours, and its proximity to the Mekong River, we hypothesised, (1) high genetic diversity of *B. pseudomallei* from Myanmar, (2) genotype overlap between Myanmar and its neighbours and (3) multiple introduction events of *B. pseudomallei* into Myanmar.

Our MLST and phylogenetic analysis demonstrated that *B. pseudomallei* from Myanmar are genetically diverse and heterogenous, with nine STs identified amongst only sixteen isolates. This finding is consistent with the high levels of genetic and ST diversity in *B. pseudomallei* from neighbouring countries including India^32^, China^33^ and Thailand^34^ and in the ancient *B. pseudomallei* population from the Northern Territory of Australia^35,36^. Five of the Myanmar STs had not been previously identified, with four being environmental isolates. Furthermore, based on phylogenetics at a local level, environmental isolates clustered by geography, suggesting geographical restriction of *B. pseudomallei* within Myanmar, as demonstrated in Australia^36–38^. However, more environmental sampling in Myanmar is necessary to elucidate whether genotype-geographical links exist and on what spatial scale. Four STs had previously been reported and are shared by neighbouring countries: ST56 ST90, ST346 and ST1371, and additional ST56 genomes were publicly available. Our phylogenetic analysis demonstrated that, despite the ST56 genomes being of Asian origin, the Myanmar ST56s are genetically distinct from those from Bangladesh and Vietnam (separated by 5,688 to 12,103 SNPs-indels) and did not all share a common ancestor, clustering in three phylogenetic locations. This represents another instance of ST homoplasy, which with increasing whole genome analyses is being recognised increasingly and which highlights the limitations of MLST, with unrecognised ST homoplasy suggesting incorrect associations between *B. pseudomallei* genotypes that confounds analysis of melioidosis epidemiology^39–42^. The genetic distance of ST56 genomes from the Australasian node suggests that ST56 may have spread from Vietnam to Myanmar and then from Myanmar on towards Bangladesh, but additional ST56 isolates are needed to confirm this hypothesis.

It is now clear that *B. pseudomallei* arrived into Myanmar from elsewhere in Asia, with all Myanmar isolates nestled within the Asian clade in our global phylogeny. Based on our global phylogenetic tree, the Myanmar isolates clustered within two distinct clades (representing two distinct populations) rather than in a single clade and so a recent common ancestor is not shared among all Myanmar isolates. This suggests that *B. pseudomallei* was introduced into Myanmar on at least two independent occasions. Based on our global phylogeny, it seems likely that *B. pseudomallei* initially arrived into Myanmar firstly from neighbours including India, Bangladesh and Malaysia and the node was well supported on our phylogeny (node shared with Myanmar isolates: strong bootstrap support 92%). With subsequent reintroductions from Thailand, Laos, Malaysia, India, Bangladesh, Pakistan, and China and the node on the phylogeny was well supported (node shared with Myanmar isolates: strong bootstrap support of 97%). In support of this our ST data showed overlap between isolates from Myanmar with isolates from Bangladesh, India, Thailand or China and so it was no surprise that Myanmar isolates grouped with isolates from those countries on the whole-genome level. Likewise, previous phylogenomic studies of *B. pseudomallei* have described repeated introduction of *B. pseudomallei* between countries bordered by the Mekong River and the Malay Peninsula^12^, with the Mekong River representing a hotspot for *B. pseudomallei* evolution and transmission throughout Southeast Asia^12^.

The observed connectivity, mode and time period of *B. pseudomallei* transmission into and within Myanmar is likely explained by severe weather events, especially flooding, geographical proximity to neighbours, human and animal migration, cultural links, prehistoric and current trading networks associated with the Mekong River. Our global phylogeny demonstrated that the Myanmar isolates located in clade 1 nearest to the tree base represent possibly the earliest introduction of *B. pseudomallei* into Myanmar, which could have been facilitated by the land bridges of Sundaland present during the last ice age (Fig. 1A). A similar theory has been postulated for *B. pseudomallei* populations in Papua New Guinea, the Sahul land bridge between Australia and New Guinea during the last ice age^43^. It is also worthy of note, however that genetic analysis of humans from Myanmar and Southwestern China (~ 6,000 individuals) demonstrated that these individuals share haplogroups (sharing a common ancestor)^44^, with individuals from China migrating to Burma likely along river valleys that connect Myanmar and China (e.g. Ayeyarwady River) around 25,000 years ago. This migration provides another potential for anthropogenically driven introduction of *B. pseudomallei* into Myanmar and fits with the period when *B. pseudomallei* was thought to have spread from Australia to Asia (between 16 and 225 thousand years ago^12^). Additionally, animal migration may have played a role in the introduction of *B. pseudomallei* to Myanmar, for example birds, which can both asymptomatically carry *B. pseudomallei* and develop melioidosis^45,46^. Myanmar has over 1,000 native bird species of which ~ 200 are migratory, so it is plausible that *B. pseudomallei* has been introduced into Myanmar by returning migratory birds. Whatever routes were responsible for the initial introduction of *B. pseudomallei* into Myanmar, the underlying genetic diversity demonstrated by our global phylogeny and extensive recombination supports the notion that it was not a recent event, with enough time elapsed since its introduction for divergence to occur. Future phylogenetic and molecular clock investigations using a larger number of Myanmar and Asian isolates will provide further clues as to the origin of *B. pseudomallei* in Myanmar.

In addition to our phylogeographic analysis, we screened the Myanmar *B. pseudomallei* assemblies against variably present virulence genes. *Burkholderia* intracellular motility factor A (BimA) is responsible for actin-based motility of *B. pseudomallei*^47^ and all Myanmar isolates contained the more common *bimA*_Bp_, which is typically identified across Asia and has been associated with pneumonia^48^. *bimA*_Bm_ has only been identified in Australian and Indian isolates and is associated with neurological melioidosis^39,48–50^. Filamentous hemagglutinin (fha) is a surface protein that functions as an adhesin^51^. Although *B. pseudomallei* contains three *fha* genes that vary genetically, it is the presence of *fhaB3* variant that has been associated with positive blood cultures and the absence of *fhaB3* with localised skin abscess^48^. *fhaB3* was variably present among the Myanmar isolates, absent from five and present in eleven, and similar variability has been noted in isolates from India, Australia and Thailand^48,49^. Lastly, we investigated the LPS types. LPS of *B. pseudomallei* is required for serum resistance, with three types being described: LPS A, LPS B and LPS B2. LPS A was dominant amongst Myanmar isolates but LPS B was also detected and prevalence of LPS B in Myanmar was higher than in Thailand^52^. Interestingly, LPS B is the most common LPS type in India, a close neighbour of Myanmar^49^.

In conclusion we have used WGS and large-scale comparative-genomics to describe for the first time the genetic diversity and phylogeography of *B. pseudomallei* in Myanmar, the country where melioidosis was originally recognised. We have demonstrated that *B. pseudomallei* from Myanmar are genetically diverse, supporting the long-term endemicity of *B. pseudomallei* in Myanmar. Phylogenomic analysis revealed that Myanmar *B. pseudomallei* cluster in the Asian clade linking to isolates from neighbouring countries, including those bordered by the Mekong River. It is thus likely that *B. pseudomallei* has been introduced into Myanmar from these close neighbours on more than one occasion. Further studies are required to elucidate the role of anthropogenic factors in both the past and evolving regional and global dispersal of *B. pseudomallei.*

## Supplementary information

Supplementary Figure S1.

Supplementary Figure S2.

Supplementary Table S1.
