# Supplementary figures and images for: Myanmar Burkholderia pseudomallei strains are genetically diverse and originate from Asia with phylogenetic evidence of reintroductions from neighbouring countries
Source: Sci Rep. 2020 Oct 1;10:16260. doi: 10.1038/s41598-020-73545-8 (PMC7530998; doi:10.1038/s41598-020-73545-8)

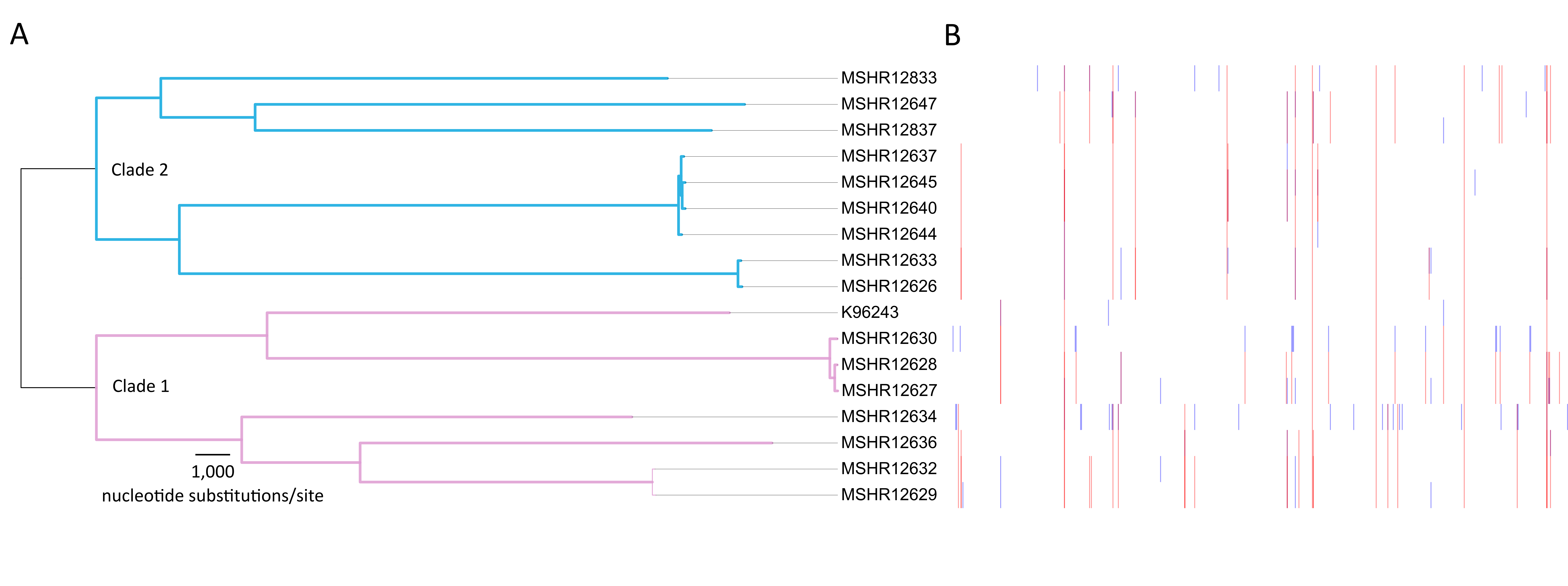

Supplement: Supplementary file 1 — Supplementary Figure S1. [file 41598_2020_73545_MOESM1_ESM.png]

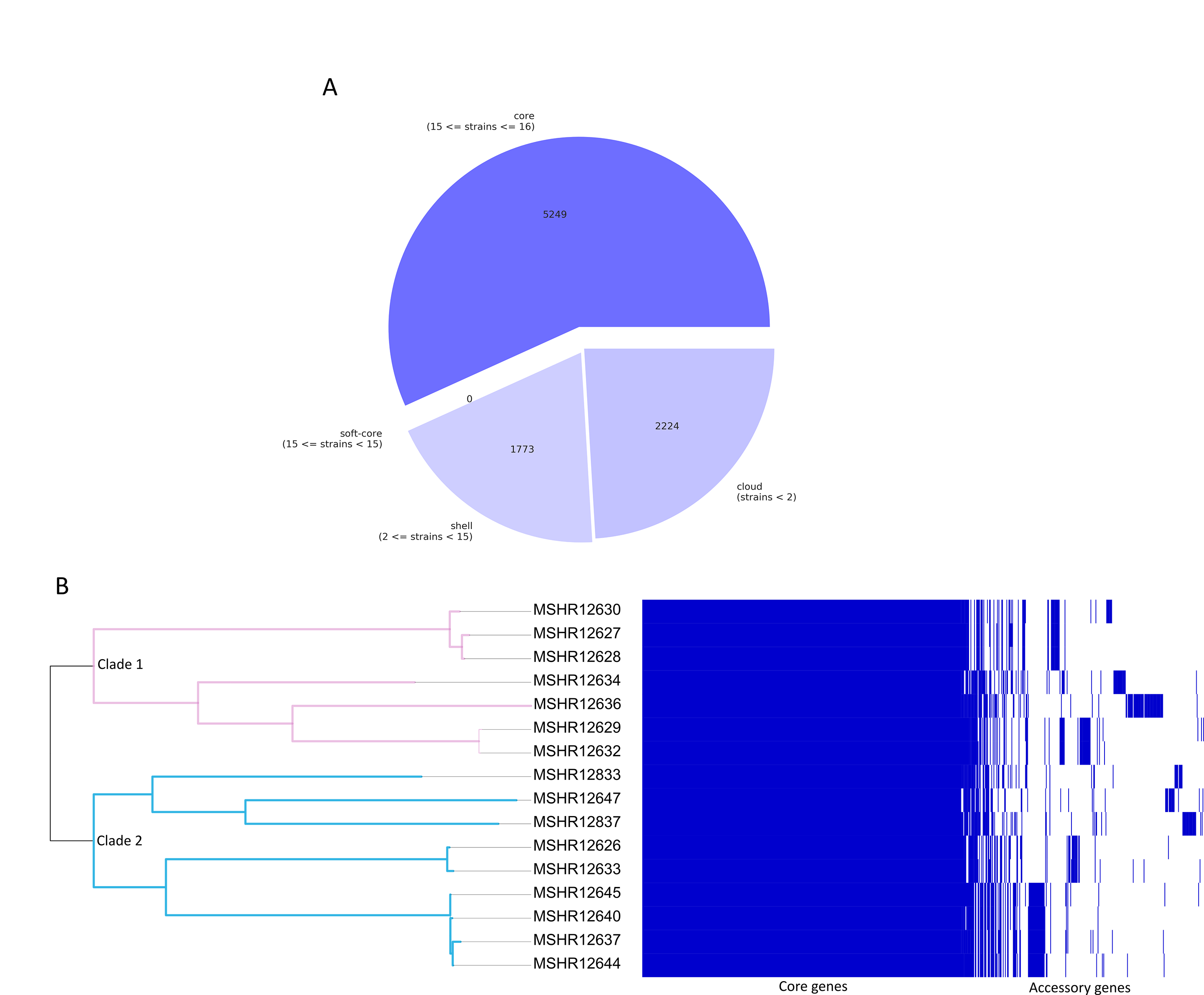

Supplement: Supplementary file 2 — Supplementary Figure S2. [file 41598_2020_73545_MOESM2_ESM.tif]
